# Nanotechnology in Advanced Drug Delivery

**DOI:** 10.1155/2011/343082

**Published:** 2011-10-25

**Authors:** Sanyog Jain, Ijeoma F. Uchegbu, Guru Betageri, Giorgia Pastorin

**Affiliations:** ^1^Centre for Pharmaceutical Nanotechnology, Department of Pharmaceutics, National Institute of Pharmaceutical Education and Research (NIPER), Sector 67, Phase X, SAS Nagar (Mohali), Punjab 160062, India; ^2^Department of Pharmaceutics, School of Pharmacy, University of London, 29 39 Brunswick Square, London WC1N 1AX, UK; ^3^Graduate College of Biomedical Sciences, Western University of Health Sciences, 309 E. Second Street, Pomona, CA 91766, USA; ^4^Department of Pharmacy, National University of Singapore, 3 Science Drive 2, Block S15, 05-PI-03, Singapore 117543

Buzzing of nanotechnology in each and every aspect of science and technology has been booming at a tremendous rate now a day. Started its journey from inorganic chemistry, this field has now even reached to aeronautical research, and a special attention has been drawn in the medical and allied braches for exploitation of the nanotech for attending the limitations of the current scenario. Carrying foreword the success of nanotechnology in field of physical, chemical, and medical sciences, it has now started revolutionizing the drug delivery sciences. The specific advantages include superior pharmacodynamics, pharmacokinetics, reduced toxicity, and targeting capability. Vehicle in the delivery sciences being critical quality attribute needs special attention for tailor made design to rationalize the formulation development; which can be successfully achieved via nanotechnology. Additionally, unique size-dependent properties of nanosystems/nanodevices offers excellent opportunities for the development of novel “point-of-care” devices and therapeutic tools. Drugs incorporated in the nanocarriers, either physically entrapped or chemically tethered, have the potential to target physiological disorder zone sparing normal cells from collateral consequences.

Targeting several molecular mechanisms, for either treatment or prevention of difficult-to-treat diseases, for the design of the various nanotechnology-based drug delivery systems is one of prime focuses of the formulation scientist at the present juncture. Gene therapies and gene-based drug delivery using nanocarriers are booming especially in case of neoplastic extravasations. Various tumor suppressor genes are identified, isolated and successfully formulated for treatment of cancer. A remarkable number of such systems have already made their pave and are under clinical trials, being expected very soon at the end user level. Besides treating these diseases, nanotechnology also offers its contributions in development of preventive measures such as vaccines. Various nanotechnological adjuvants have been evaluated for their capabilities to deliver vaccine subunits without compromising the immunogenicity for successful design and development of vaccine delivery systems. Furthermore, regeneration ability of the visceral organs such as liver has also been evaluated using nanocarriers, and the postulations are on their way stating the enhanced cytoactivity of the transplanted cells when cultured in nanocarriers. 

The pharmacokinetic profile, especially transportation capabilities, of the drug substances have been greatly modified by incorporation in nanodrug delivery system. These include enhanced accommodation for targeting moieties such as chaperones and alteration in release rates comprising of controlled release and site-specific delivery by use of molecular engineering techniques. Additionally, encapsulation of the drug substances in various polymeric and inorganic composites has also been evaluated for their rationalizing the drug delivery systems. Such encapsulations are generally made for protecting the biologically active protein and peptide-based drug compounds from the detrimental effects of biological fluids. Newer nanoprodrug approaches have also been applied, which has posed enhanced therapeutic efficacy along with superior circulation time. 

Emerging methodologies for formulation of nanodrug delivery systems include newer versions of the top-down and bottom-up approaches. Additionally allied technologies such as atomization and pressurization have come in to play to facilitate the preparation of nanotechnological carriers. One such comprises a novel method of atomization, namely, electrohydrodynamic atomization used in electrospraying method. Pressurization techniques such as high hydrostatic pressure technology for encapsulation of genes into polymeric nanomaterials have recently been studied for their efficacy in delivering the biologically active compounds. These novel technologies offer advantages by eliminating the usage of toxic cationic polymers and chemical tethers further replacing them simple yet effective hydrogen bonding. Such advantages and simplifications of the process have already given their imminent revolutions in the field of drug delivery. 

With these advancements in the novel nanocarriers and their applicability, the analytical tools are also not lagging behind. In order to cope up with this and stand aside, newer evaluation methodologies are already in move of their development. These include scanning probe microscopy, more specifically atomic force microscopy and scanning tunneling microscopy, which have great capabilities for molecular and submolecular characterizations. 

Furthermore, various platform technologies at the nanoscale, often referred as nanoplatforms, have been coming in to play and booming for widespread applications of these cutting edge technologies for its applicability at end users. Basically, the common ones in the race include the nanocrystals (nanopure, nanoedge, Dissocubes), nanomaterials (fullerenes, carbon nanotubes, and nanoparticles), nanomedicines, molecular self-assemblies (self-assembled monolayers, supramolecular assemblies and DNA nanotechnologies), nanoelectronics (*in silico* technologies), and so on to name a few. The extension and appliance will be the state-of-art for future research. 

Finally, the common undeniable opinion highlighted in this issue is that, although it is too early to say whether these nanocarriers will wean the world from its current limitations, or monumentally backfire to cause harm, a deep understanding of the various mechanisms underneath the reported findings will favor great discoveries, even at the nanoscale. 

In nutshell, efforts are extensively made for utilizing the nanotechnology concepts for advancements in the current drug delivery sciences and, further to this, the fruitfulness of the efforts has been achieved which is well reflected in this present issue. 

*Sanyog Jain* *Sanyog Jain* *Ijeoma F. Uchegbu* *Ijeoma F. Uchegbu* *Guru Betageri* *Guru Betageri* *Giorgia Pastorin* *Giorgia Pastorin*

